# HYPOTHALAMIC MICROSTRUCTURE IN OLDER ADULTS IS ASSOCIATED WITH CORTICAL ATROPHY AND NEUROCOGNITION

**DOI:** 10.1093/geroni/igad104.0131

**Published:** 2023-12-21

**Authors:** Sandra Aleksic, Roman Fleysher, Noa Tal, Timothy Darby, Erica Weiss, Nir Barzilai, Michael Lipton, Sofiya Milman

**Affiliations:** Albert Einstein College of Medicine, Bronx, New York, United States; Albert Einstein College of Medicine, Bronx, New York, United States; Cedars-Sinai Medical Center, Los Angeles, California, United States; Albert Einstein College of Medicine, Bronx, New York, United States; Montefiore Medical Center/ Albert Einstein College of Medicine, Bronx, New York, United States; Albert Einstein Clollege of Medicine, Bronx, New York, United States; Albert Einstein College of Medicine, Bronx, New Jersey, United States; Albert Einstein College of Medicine, Bronx, New York, United States

## Abstract

The hypothalamus is emerging as an important regulator of aging. Hypothalamic inflammation in mice shortens lifespan and accelerates neurocognitive decline. However, the role of the hypothalamus in systemic and neurocognitive aging in humans is unresolved. We studied the cross-sectional relationships between hypothalamic microstructure, cortical atrophy and neurocognition in community dwelling older adults (n=124, median age 78.7 (IQR 74.3-83.4) years, 58% women). Hypothalamic microstructure was assessed using magnetic resonance imaging (MRI)-diffusion tensor imaging (DTI) parameters that included mean diffusivity (MD) and fractional anisotropy (FA), obtained using a novel automated image processing pipeline. Greater MD and lower FA were indicative of compromised microstructural integrity. Neurocognitive assessments included a battery of 11 tests that were used to quantify standardized domain-specific (executive, memory, language, attention and visuomotor) and overall neurocognitive scores. Age was positively associated with hypothalamic MD (beta=0.037 SD per year of age, p=0.005). In linear models adjusted for age, sex, education, white matter hypointensities, and total intracranial volume, hypothalamic MD was negatively associated with cortical thickness in the cingulate, frontal, and parietal lobes (betas -0.07, -0.06, -0.07 respectively, all FDR-adjusted p≤0.01). On the other hand, in a multivariable adjusted model that included cortical thickness, hypothalamic FA was positively associated with the overall and all domain-specific neurocognitive scores (betas ranged 0.14 - 0.18, FDR- adjusted p values< 0.05). Hypothalamic microstructure is independently associated with cortical atrophy and neurocognition in the aging brain. Longitudinal studies can establish temporality and potential mechanisms by which hypothalamic dysfunction may accelerate systemic and neurocognitive aging in humans.

